# Colonoscopy Screening Behaviour and Associated Factors Amongst First-Degree Relatives of People with Colorectal Cancer in China: Testing the Health Belief Model Using a Cross-Sectional Design

**DOI:** 10.3390/ijerph17144927

**Published:** 2020-07-08

**Authors:** Yang Bai, Cho Lee Wong, Xiaolin Peng, Winnie K. W. So

**Affiliations:** 1 The Nethersole School of Nursing, The Chinese University of Hong Kong, Hong Kong, China; jojowong@cuhk.edu.hk (C.L.W.); winnieso@cuhk.edu.hk (W.K.W.S.); 2 Shenzhen Nanshan Center for Chronic Disease Control, Shenzhen 518054, China

**Keywords:** colorectal cancer, first-degree relatives, colonoscopy, cancer screening, the health belief model

## Abstract

Colonoscopy is the best screening choice for at-risk persons, because it offers prevention through the removal of preneoplastic lesions in addition to early detection. This study aims to report the participation rate of colonoscopy screening and examine its associated factors amongst Chinese first-degree relatives of people with colorectal cancer based on the health belief model (HBM). A cross-sectional study was conducted in Shenzhen, China from March to May 2019. Demographic characteristics, family history, variables derived from the HBM and colonoscopy screening behaviours were measured through online surveys as the independent variables of interest. A total of 186 online surveys were returned, with a final response rate of 57.0%. The participation rate of colonoscopy was 15.6%. Univariate analysis (independent *t*-test/chi-square test/Fisher test) was applied first to identify the candidate independent variables. Then, multivariate logistic regression was used to examine the association between independent variables and uptake of colonoscopy. Perceived barriers and cues to action were identified as factors associated with undergoing colonoscopy. The participation rate of colonoscopy in the study population was low. Health communication to promote colonoscopy screening for the Chinese at-risk population should include components in reducing barriers to colonoscopy tests, family history information and health professional recommendations on screening. Future studies with large sample size are suggested to examine perceived susceptibility, fatalism and other characteristics considering family history (treatment and outcome of patients) and their potential impacts on cancer screening behaviours for Chinese at-risk populations due to family history.

## 1. Introduction

Colorectal cancer (CRC) has been identified as one of the most common malignancies, with over 1.8 million new CRC cases and 881,000 deaths estimated in 2018 [1]. With the economic development and changes in lifestyle, a steady increase in CRC incidence was observed in China over the past three decades, revealing an increasing rate from 14.25 per 100,000 in 1990 to 25.27 per 100,000 in 2016 [2]. Family clustering is a typical feature of this disease, with approximately 25% of CRC cases having a first-degree relative (FDR) diagnosed with CRC [3]. Compared with the general population, FDRs of people with CRC have a risk of developing CRC from twofold to threefold [4].

Previous studies have reported that FDRs of people with CRC can benefit from the removal of polyps through endoscopic screening, which decreases CRC incidence by 75–90% [5,6,7]. The National CRC Screening Guideline in China is consistent with international ones to recommend individuals with one FDR with CRC at <60 years or with two or more FDRs with CRC at any age a colonoscopy for at the age of 40 years or 10 years earlier than the youngest CRC case in the family, with an interval of 5five years [4,8]. 

Japan, Australia, France and the UK had started pilot screening programmes for CRC by 2002 [9]. A total of 22 countries have commenced screening programmes for CRC by 2015. The population-based Cancer Screening Programme in China was promoted in 2012, with partially or totally free colonoscopy. Although at-risk populations could benefit from the screening for CRC by decreasing potential risks, the colonoscopy screening uptake rates amongst the families of patients are similar to the average population with a low rate of 40% [10]. The situation in China is even worse, because less than 20% of people received recommended colonoscopy, despite it has already been covered by insurance [11]. 

Several studies were conducted to examine the factors associated with the participation in CRC screening amongst FDRs of people with CRC, such as barriers to screening, demographic and lifestyle characteristics [12,13,14,15,16]. Demographic characteristics were the most frequently examined factors, with age, education level and insurance as identified predictors to participate in colonoscopy screening [12,13,14,15]. A review conducted by Tan et al. summarised eight qualitative studies [16], which reported that important barriers to CRC screening tests include a fear of cancer diagnosis, discomfort, embarrassment, cost of test and accessibility to health care resources and the lack of awareness of the increased risk of disease development. Dietary habits, physical activities and regular physician examinations were reported as lifestyle factors related to screening behaviours amongst FDRs of people with CRC [12,15]. Additionally, variables from the health behaviour change theory are crucial in understanding the mechanism underlying preventive behaviours and preparing interventions designed to enhance the adaptation of health behaviours. 

The health belief model (HBM) is a conceptual framework to explain and predict the health behaviour. This model posits that people are likely to engage in health prevention behaviours if they have high perceptions of susceptibility, severity, benefits and self-efficacy and a low level of barrier perceptions. In addition to the five aforementioned factors, health behaviours are also influenced by cues to action (events or people that act as triggers for the action). The HBM has been widely used in predicting and explaining cancer screening behaviours [17,18,19]. Variables derived from the HBM have been identified as significant targets when developing communication interventions to promote screening for the at-risk population [20]. 

Only one study examined the associated factors of screening colonoscopy based on the HBM by using the Turkish version of the Champion’s Health Belief Model Scale (CHBMS) and indicated that perceived benefits and health motivations were significantly associated with colonoscopy screening [15]. The CHBMS was developed and adapted to identify beliefs related to CRC based on the HBM [21,22]. However, important extensions to the original HBM—namely, cues to action and self-efficacy—were not measured on this scale. Additionally, such evidence regarding determinants of colonoscopy screening behaviours based on the theoretical framework of the HBM was rarely reported in the Chinese at-risk population. Therefore, this study aims to report the participation rate of colonoscopy screening and examine the variables associated with participation in colonoscopy screening based on the HBM amongst Chinese at-risk populations due to family history.

## 2. Materials and Methods 

### 2.1. Study Design and Participants

**Figure 1:**
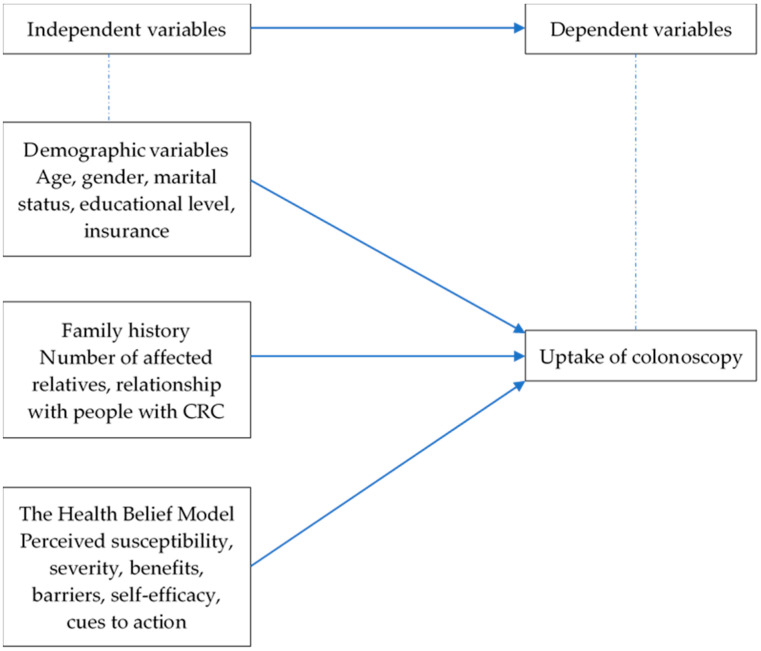
Independent and dependent variables. CRC: colorectal cancer.

A cross-sectional survey was conducted from March to May in 2019 in Shenzhen, China. A convenience sample of FDRs of people with CRC was recruited through the Nanshan Chronic Disease Management Centre, Nanshan Hospital and 23 community centres. People were eligible if they meet the following criteria: (1) 40–75 or 10 years of age before the relative was diagnosed, (2) individuals with one FDR with CRC at <60 years of age or with two or more FDRs with CRC at any age, (3) have access to WeChat or through close family members living in the same household and (4) can read and speak Chinese. The following individuals were excluded: (1) have a history of cancer or inflammatory bowel disease and (2) with doctor-diagnosed psychiatric illness. Online questionnaires were sent to consenting participants through WeChat. According to the rule of thumb—namely, 10 cases per variable—a sample size larger than 130 was sufficient for 13 independent variables (age, gender, marital status, educational level, insurance, number of affected relatives, relationship with people with CRC, perceived susceptibility, perceived severity, perceived benefits, perceived barriers, self-efficacy and cues to action) to provide accurate inferences in logistic regression [23]. The independent variables and dependent variable are shown in Figure 1.

### 2.2. Measures 

#### 2.2.1. Colonoscopy Screening Behaviour

There is not a national electronic medical records system in China to see if a participant has received a colonoscopy. It is also difficult to ask participants to provide their medical records of colonoscopy in the past 10 years. Therefore, this study used the self-report method to evaluate the previous colonoscopy screening behaviours, with No (0)/Yes (1) as the response format.

#### 2.2.2. Perceived Susceptibility, Severity, Benefits and Barriers

The perceived susceptibility of developing CRC, perceived severity of CRC, perceived benefits of colonoscopy and perceived barriers to colonoscopy were measured by the simplified Chinese version of a 38-item Revised Colorectal Cancer Perception and Screening Instrument (RCRCPS) (see in Appendix A) [24,25,26]. This scale, which has four subscales to measure the four aforementioned psychosocial variables, was developed on the basis of the HBM. Each item is rated using a five-point Likert-type scale (1 = strongly disagree to 5 = strongly agree). The score for each subscale was computed by averaging the corresponding items. A high score in each subscale indicates a high level of perception. The internal consistency of the simplified Chinese version of the RCRCPS was validated with Cronbach’s alpha, ranging from 0.74 to 0.87 [24]. The results of construct validity support the application of the original four-factor HBM model for the 38-item RCRCPS.

#### 2.2.3. Self-efficacy

Self-efficacy to receive colonoscopy was measured by the four-item simplified Chinese version of the Self-efficacy Questionnaire (see in Appendix B) [24]. This questionnaire was developed by Wagner et al. (2009) to assess the self-efficacy for participating in CRC screening. The questionnaire has been translated into simplified Chinese and adapted for a colonoscopy test, indicating satisfactory reliability, with a Cronbach’s alpha of 0.77 [24]. The items are rated on a five-point scale (1 = strongly disagree to 5 = strongly agree). The score for self-efficacy was computed by averaging the corresponding items (ranging from 1 to 5). The total score ranged from 4 to 20, with a high score indicating a high self-efficacy to participate in CRC screening.

#### 2.2.4. Cues to Action

Cues to action amongst the FDRs of people with CRC are operationally defined in this study as cues that prompt colonoscopy screening behaviours. Three types of action cues (history of CRC amongst family members, recommendations of healthcare professionals and health insurance) were involved and measured by the cues to action questionnaire in this study (see in Appendix C). The items are rated on a five-point scale (1 = strongly disagree to 5 = strongly agree). The score for cues to action was computed by averaging the corresponding items (ranging from 1 to 5), and a high score indicated a high cue to action.

#### 2.2.5. Demographic and Family History

Based on previous studies [12,13,14,15], demographic characteristics, including potential factors associated with CRC screening behaviours—namely, age, gender, marital status, educational level and insurance—were collected. A family history of CRC was also collected. Information regarding the family history, including relationship to patients and the number of patients affected by CRC, was recorded. 

### 2.3. Ethical Considerations

Ethical approval was obtained from the Joint Chinese University of Hong Kong-New Territories East Cluster Clinical Research Ethics Committee (CREC Ref. No.: 2018.368) and the Nanshan Chronic Disease Management Centre (ll20180013). Participants were assured of the confidentiality of personal information. Online questionnaires with responses of participants did not bear names but were identified by codes.

### 2.4. Data Analysis

Descriptive statistics were used to describe demographic characteristics, family history and HBM variables with means and standard deviations (SD) for continuous variables and frequency (%) for categorical variables. The dependent variable (colonoscopy screening behaviour) was dichotomised to the following: (1) have had a colonoscopy and (2) have not had a colonoscopy. Sociodemographic characteristics, family history and HBM variables were independent variables of interest. Univariate analysis was examined on the association between each of the independent variables and the uptake of colonoscopy by using an independent *t*-test (continuous variable) and chi-square/Fisher test (categorical variable). The Fisher exact probability test was used when the expected frequencies were less than six in each cell [27]. Variables considered statistically significant (*p* < 0.25) in the univariate analysis were selected as candidate independent variables into a multivariable logistic regression. The value of 0.25 is recommended by a leading expert in logistic regression analysis, Hosmer et al. [23], which can largely avoid ignoring independent variables weakly associated with dependent variables alone but become significant when taken together. The results of the multivariable logistic regression model are presented by the odds ratios (OR) and their associated 95% confidence intervals (CI). *p* < 0.05 was considered statistically significant in the multivariate logistic regression. Data were analysed using IBM SPSS statistics (version 25, SPSS Inc., Chicago, IL, USA).

## 3. Results

A total of 186 of the 328 online surveys were returned, with a final response rate of 56.7%. 

By using GPower 3.1 (Heinrich Heine University, Düsseldorf, Germany), such a sample size (n = 186) enabled detection with an odds ratio as small as 1.75, with 80% power at a one-sided 2.5% level of significance for independent variables.

### 3.1. Demographic and Family History Characteristics 

Table 1 presents the characteristics of the sample. The mean age of the FDRs was 49.62 (SD, 9.12), with a range of 28–70 years. The majority of involved FDRs were married (n = 174, 93.5%), and 109 (58.6%) were female. Most of the participants (n = 171, 91.9%) were covered by health insurance. More than 70% of the FDRs received secondary education. Considering family history, only four (2.2%) FDRs had two relatives with CRC. Most of the FDRs involved in this study were children of original cancer cases. 

### 3.2. HBM Variable

The mean scores for each subscale were 2.89 (SD, 0.64), 3.19 (SD, 0.67), 4.20 (SD, 0.50), 2.66 (SD, 0.56), 4.06 (SD, 0.44) and 3.98 (SD, 0.39) for perceived susceptibility, perceived severity, perceived benefits, perceived barriers, self-efficacy and for cues to action, respectively. 

### 3.3. Screening Behaviours

A total of 29 (15.6%) participants reported undergoing colonoscopy (Table 1).

### 3.4. Factors Associated with Having a Colonoscopy 

The results of the univariate analysis of sociodemographic characteristics, family history and the HBM variables associated with the participation of colonoscopy are presented in Table 1. Eight variables—namely, gender, education level, insurance, number of affected relatives, perceived benefits, perceived barriers, self-efficacy and cues to action—had *p*-values less than 0.25. All eight variables were included in the multivariable logistic regression. Amongst these variables, perceived barriers (OR, 0.325; 95% CI, 0.129, 0.816; *p* = 0.017) were negatively associated, whilst cues to action (OR, 3.137; 95% CI, 0.916, 10.087; *p* = 0.014) were positively associated with the likelihood of having a colonoscopy. However, the other variables became insignificant (Table 2).

## 4. Discussion

### 4.1. Screening Rate of Colonoscopy 

The self-reported participation rate of colonoscopy screening in the present study is 15.6%, which is lower than the rates reported in the USA, Canada, Australia and some parts of Europe (26%–54%) [10]. This low rate could be attributed to two possible reasons. Firstly, the population-based screening programme was released relatively late compared with that of other countries. As previously introduced, the population-based screening programme for CRC in China was promoted almost 10 years later than the earliest countries, such as Japan and the UK. The Chinese general public may not have sufficient literacy on cancer screening, because screening programmes emerged only in recent years [28]. Secondly, despite the usage of conventional strategies (for example, brochures, posters and recommendations of community health workers) to publicise the screening programme, a low public awareness and acceptance of screening programmes were consistently reported in Chinese populations [29,30]. This finding may indicate the limited capacity of conventional strategies to promote colonoscopy screening and the need of additional active interventions. Understanding changeable psychosocial variables associated with screening behaviours is crucial to helping health professionals develop interventions to promote the screening rates of colonoscopy. However, such evidence is rarely reported in a Chinese at-risk population due to family history. This study addressed this gap by identifying factors associated with colonoscopy screening behaviours amongst Chinese FDRs of people with CRC.

### 4.2. Factors Associated with Colonoscopy Screening Behaviours

#### 4.2.1. Sociodemographic Variables

In the present study, no relationship was identified between sociodemographic variables and undergoing colonoscopy. The findings were inconsistent with previous studies, which reported age, education level and insurance as identified predictors for colonoscopy screening [12,13,14,15]. However, the findings were consistent with a study conducted for community-dwelling Chinese older people [31]. The inconsistent results may be explained by the nature of cross-sectional studies or the mediation effects of some factors associated with CRC but not included for examination [32]. The CRC screening programme was promoted relatively late, and this study is the first to examine association factors of colonoscopy screening amongst Chinese FDRs of people with CRC. Further studies are needed to examine the relationship between these variables in the context of Chinese culture.

#### 4.2.2. Family History Variables

Relationships with affected patients were not correlated with colonoscopy screening behaviours in this study; this finding is consistent with a study conducted for FDRs in Turkey [15]. Additionally, the results of the multivariate analysis showed no relationship between the number of affected patients and the likelihood of having a colonoscopy. The insignificant results of the number of affected relatives with CRC were inconsistent with those of previous studies [15,33]. Such discrepancies on family history variables have been attributed to the proportion of the provided sample. Only 2.2% of participants have more than one FDR, which may underestimate the strength of the association. Thus, a large sample size with a rich percentage of people with different characteristics is suggested for future studies.

#### 4.2.3. HBM Variables

Amongst HBM variables, the results showed that two variables—namely, perceived barriers and cues to action—were related to the participation of colonoscopy screening. This finding is consistent with a study for FDRs in Israel, in which barriers and cues to action were reported as the determinants of participation in colonoscopy screening [12].

The obtained findings suggested that colonsocpy screening promotion interventions for Chinese FDRs should include components of addressing barriers to colonoscopy. Sixteen barrier items from the perceived barriers subscale of RCRCPS were used to measure perceptions regarding barriers to colonoscopy screening. The highest percentage of agreement (agree/ strongly agree) in the present study were responses to do not know how to schedule screening (46.9%), the exam may be painful (37.7%), afraid to find something wrong with me (33.0%) and do not understand what will be done in the test (31.4%). Compared with barriers identified in Western countries that focused on the psychological aspects, such as anxiety, embarrassment and vulnerability [34], the important barriers identified in the present study focused on knowledge regarding the screening test. The possible reason was that Chinese people may have limited literacy regarding CRC screening at this early stage of CRC screening promotion. Thus, screening-promotion activities should focus on what is conducted during colonoscopy, especially the assistance of participants in understanding the procedure of colonoscopy, including the preprocedural consultation, colonoscopy-tolerance evaluation, appointment, bowel preparation (bowel cleansing and diet control), sedation to relieve pain from the procedure, polyps removal, pathological examination for identified polyps or cancers and postprocedure observation.

Internal (family history and insurance) and external (recommendation of health professionals) cues to action were examined in this study. Physicians’ recommendations were consistently reported to influence CRC screening behaviours among the Chinese population [28]. The present study adds evidence that it is also an associated factor among the at-risk population in China. Health information regarding family history and cancer screening provided by healthcare professionals may be an effective approach to promote colonoscopy screening amongst people at increased risk due to family history.

Although the perceived susceptibility of developing CRC was identified as a significant factor in a previous study, which summarised 22 quantitative studies in the Chinese population [28], an insignificant result was revealed in the present study. The discrepancy may be attributed to the different targets of the screening tests. Unlike the review summarising the evidence for any type of CRC screening test, the present study focuses on colonoscopy. The procedure of colonoscopy is more invasive, complex and labour-intensive than that of other stool-based bowel screening tests [34]. The data analysis involving colonoscopy and other screening tests may introduce barriers to the results.

The predicted relation of the perceived severity of CRC found in this study is consistent with the review conducted by Kiviniemi et al. [32]. This review summarised 81 studies examining the relationship between psychosocial constructs and CRC screening behaviours. The majority of studies in this review did not support the theory-derived relation between severity and screening behaviours. The perceived severity of CRC may not work when discriminating between people who have received CRC screening and those who have not. Therefore, longitudinal studies are needed to test the assumption further.

Perceived benefits and self-efficacy have been reported as predictors of CRC screening in previous studies [32]. However, no significant association on benefits and self-efficacy was found in the present study. Leung et al. [31] found similar insignificant results on perceived benefits and self-efficacy amongst community-dwelling old Chinese people in Hong Kong. One possible reason for this interesting result in the Chinese population may be fatalistic beliefs. The belief that health and illness are predetermined and beyond the control of an individual is fatalistic [35]. This construct has been identified as a specific factor influencing health beliefs in the Chinese population [36]. Such beliefs in Chinese culture may hinder health-prevention behaviours, despite the high levels of benefits and self-efficacy found in the study sample. However, the effects of fatalism on health beliefs in the Chinese population remain unclear. Additional studies are suggested to explore the mechanisms underlying how fatalistic beliefs influence health beliefs and cancer screening behaviours in the Chinese population to enhance the influence of health communication on cancer prevention and screening.

### 4.3. Strengths of the Study

The FDRs of people with CRC are healthy people who are difficult to identify in health institutions directly. The traditional recruitment approach focuses on identifying FDRs through CRC cases by telephone. Poor recruitment has always been reported in family trials owing to inadequately documented family histories and intensive contacts to recruit one subject [37]. Accordingly, in addition to the traditional approach, we applied herein three other methods through the current CRC screening programme to identify FDRs, clinical settings to identify FDRs who are taking care of hospitalised CRC patients and recruitment posters and messages in community centres. After utilising these recruitment approaches through trustworthy sources, a response rate of 57% was achieved in this study.

### 4.4. Limitations of the Study

A list of FDRs for random sampling would have not been feasible for this population, because information on family histories is not well-documented. The participants were conveniently recruited from one district in Shenzhen, a developed city in China. The use of the convenience sampling method may reduce the representativeness of the sample and the generalisation of the study findings to less-developed or rural areas in China. This study may only have representativeness of large and developed districts, such as Beijing, Shanghai and Guangzhou. Some confounders such as treatment and outcomes of CRC patients, which have been reported as associated factors of CRC screening behaviours amongst FDRs of people with CRC [15], were not collected in this work. We used a self-reporting method to evaluate the uptake of colonoscopy, and this method was likely to have been influenced by reporting bias.

## 5. Conclusions

This is the first study to report the participation rate of colonoscopy and examined sociodemographic, family history variables and modifiable factors from the HBM for the likelihood of receiving colonoscopy amongst Chinese FDRs of people with CRC. The participation rate of colonoscopy remains low in the at-risk population. Perceived barriers and cues to action were the identified factors associated with the participation of colonoscopy screening. Colonoscopy screening promotion programmes for at-risk populations due to family history should include the following components: (1) information to help participants understand the colonoscopy procedure, (2) family history information and (3) health professionals’ recommendations on screening. Future studies are suggested to examine cultural beliefs (e.g., fatalism) and other characteristics of family history, such as the treatment and outcomes of patients, as well as their potential impacts on cancer screening behaviours for Chinese at-risk populations due to family history.

## Figures and Tables

**Table 1:** Social demographic, family history and the health belief model variables and their relationship with undergoing colonoscopy (n = 186).

Independent Variables	All (n = 186)	Mean ± SD or Frequency (%)	Not ever Had a Colonoscopy (n = 157)	*p*-Value
		Ever Had a Colonoscopy (n = 29)		
**Demographic Characteristics**				
Age ^†^ (range: 28–70)	49.62 ± 9.12	49.69 ± 9.127	19.60 ± 9.210	0.961
Gender				
Female	109 (58.6)	20 (18.3)	89 (81.7)	0.217 *
Male	77 (41.4)	9 (11.7)	68 (88.3)	
Marital status				
Married	174 (93.5)	27 (15.5)	147 (84.5)	1.000
Single/Divorced/Widow	12 (6.5)	2 (16.7)	10 (83.3)	
Educational level				
Secondary or less (≤12 years)	140 (75.3)	18 (12.9)	122 (87.1)	0.073 *
Tertiary or above (>13 years)	46 (24.7)	11 (23.9)	35 (76.1)	
Insurance				
No	15 (8.1)	4 (2.7)	11 (97.3)	0.249 *
Yes	171 (91.9)	25 (14.6)	146 (85.4)	
**Family history**				
Number of affected relatives				
1	182 (97.8)	27 (14.5)	155 (85.5)	0.115 *
2	4 (2.2)	2 (50.0)	(50.0)	
Relationship with people with CRC				
Children	162 (87.1)	26 (16.0)	136 (84.0)	1.000
Siblings	24 (12.9)	3 (12.5)	21 (87.5)	
**The Health Belief Model variables**				
Perceived susceptibility	2.89 ± 0.64	2.97 ± 0.67	2.88 ± 0.63	0.476
Perceived severity	3.19 ± 0.67	3.11 ± 0.64	3.20 ± 0.68	0.517
Perceived benefits	4.20 ± 0.50	4.34 ± 0.45	4.18 ± 0.51	0.099 *
Perceived barriers	2.66 ± 0.56	2.37 ± 0.66	2.72 ± 0.52	0.002 *
Self-efficacy	4.06 ± 0.44	4.25 ± 0.43	4.01 ± 0.44	0.010 *
Cues to action	3.98 ± 0.39	4.20 ± 0.39	3.94 ± 0.37	0.001 *

Note: SD, standard deviation; OR, odds ratio; CI, confidence interval; NRCMI, new rural cooperative medical insurance; BSMI, basic social medical insurance; FBOT, faecal blood occult test and CRC, colorectal cancer; ^†^ mean (standard deviation)—otherwise, as frequency (%). * *p* < 0.25.

**Table 2:** Factors associated with the participation of colonoscopy screening (received colonoscopy or not received colonoscopy).

Independent Variables	Multivariable Analysis			
	OR	95%CI		*p*
		Lower	Upper	
Demographic Characteristics				
Gender				
Female	1			
Male	0.550	0.213	1.420	0.216
Educational level				
Secondary or less (≤12 years)	1			
Tertiary or above (>13 years)	2.133	0.799	5.692	0.130
Insurance				
No	1			
BSMI	0.321	0.80	1.290	0.109
Others	0.119	0.11	1.294	0.080
Family history				
Number of affected relatives				
1	1			
2	4.777	0.587	38.896	0.144
Health Belief Model variables				
Perceived benefits	1.239	0.521	2.943	0.628
Perceived barriers	0.325	0.129	0.816	0.017 **
Self-efficacy	1.132	0.348	3.676	0.837
Cues to action	3.137	0.916	10.087	0.014 **

Note: SD, standard deviation; OR, odds ratio for significant factors obtained from the univariate analysis with *p*-value < 0.25; CI, confidence interval and BSMI, basic social medical insurance; ** *p* < 0.05.

## Appendix A

**Table A1:** The Revised Colorectal Cancer Perception and Screening Instrument.

	Strongly Disagree	Disagree	Neutral	Agree	Strongly Agree
C1. It is extremely likely that I will get CRC.	1	2	3	4	5
C2. My chances of getting CRC in the next few years are great.	1	2	3	4	5
C3. I feel I will get CRC sometime in my life.	1	2	3	4	5
C4. Developing CRC is currently a possibility for me.	1	2	3	4	5
C5. I am concerned about the likelihood of developing CRC in the near future.	1	2	3	4	5
C6. The thought of getting CRC scares me.	1	2	3	4	5
C7. When I think of CRC, I feel nauseated.	1	2	3	4	5
C8. If I had CRC my career (life) would change.	1	2	3	4	5
C9. When I think of CRC my heart beats faster.	1	2	3	4	5
C10. CRC would endanger my marriage (relationship).	1	2	3	4	5
C11. CRC is a hopeless disease.	1	2	3	4	5
C12. My feelings about myself would change if I got CRC.	1	2	3	4	5
C13. I am afraid to even think about CRC.	1	2	3	4	5
C15. Problems I would experience from CRC would last a long time.	1	2	3	4	5
C16. If I got CRC, it would be more serious than other disease	1	2	3	4	5
C17. If I got CRC my whole life would change.	1	2	3	4	5
C18. CRC can be found early if screening.	1	2	3	4	5
C19. Treatment not as bad if screening.	1	2	3	4	5
C20. Best way to find smaller cancer if screening.	1	2	3	4	5
C21. Screening decreases chance of dying of CRC.	1	2	3	4	5
C22. Screening doing something to take care of myself.	1	2	3	4	5
C23. CRC screening is embarrassing.	1	2	3	4	5
C24. I am afraid I will find out there is something wrong with me.	1	2	3	4	5
C25. I am afraid to have CRC screening because I don’t understand what will be done in the test.	1	2	3	4	5
C26. I don’t know how to go about scheduling CRC screening.	1	2	3	4	5
C27. I cannot remember to schedule an appointment for CRC screening.	1	2	3	4	5
C28. Having CRC screening would take too much time.	1	2	3	4	5
C29. CRC screening exams may be painful.	1	2	3	4	5
C30. People doing CRC screening may be rude.	1	2	3	4	5
C31. Having CRC screening would expose me too much radiation.	1	2	3	4	5
C32. It is difficult to get transportation to get CRC screening.	1	2	3	4	5
C33. I have other problems that are more important than getting CRC screening.	1	2	3	4	5
C34. CRC screening would interfere with my activities.	1	2	3	4	5
C35. Having CRC screening costs too much money.	1	2	3	4	5
C36. Colonoscopy may induce body damage	1	2	3	4	5
C37. Colonoscopy may make me feel uncomfortable	1	2	3	4	5
C38. I am scared of colonoscopy	1	2	3	4	5

## Appendix B

**Table A2:** Self-efficacy Questionnaire.

	Strongly Disagree	Disagree	Neutral	Agree	Strongly Agree
1. If I received the FOB test, I would feel able to complete it	1	2	3	4	5
2. Completing the FOB test makes sense to me	1	2	3	4	5
3. The FOB test is feasible	1	2	3	4	5
4. If I received an invitation for additional testing I would accept it	1	2	3	4	5

## Appendix C

**Table A3:** Cues to Action Questionnaire.

	Strongly Disagree	Disagree	Neutral	Agree	Strongly Agree
1. If your family was diagnosed with colorectal cancer, will you receive a screening colonoscopy?	1	2	3	4	5
2. If your insurance covered the colonsocpy, will you receive this test?	1	2	3	4	5
3. If the healthcare professionals recommend you to received screening colonoscopy, will you received this test?	1	2	3	4	5
